# Outcomes of A Brain Health Self-Management Coaching Intervention

**DOI:** 10.1093/geroni/igaf122.607

**Published:** 2025-12-31

**Authors:** Kush Kinariwala, Sarah Ross, Sara Murphy, Matthew Smith

**Affiliations:** University of North Texas Health Science Center, Fort Worth, Texas, United States; University of North Texas Health Science Center, Fort Worth, Texas, United States; University of North Texas Health Science Center, Fort Worth, Texas, United States; Texas A&M University, College Station, Texas, United States

## Abstract

Research suggests modifiable brain healthy lifestyle behaviors account for one-third of Alzheimer’s Disease and related dementia (ADRD) diagnoses. While brain-healthy lifestyle choices (e.g., diet, exercise, sleep, social engagement) can help reduce the risk of developing ADRD, individuals often lack the knowledge and support needed to identify, implement, and maintain the necessary changes. Adults without ADRD were recruited to participate in an in-person three-month brain health coaching intervention hosted by certified health and wellness coaches in a clinical context. Brain health coaching targeted the Seven Pillars of Brain Health: nutrition, exercise, social engagement, cognitive activity, sleep, outlook and mindfulness, and general health. Over a three-month period, participants met with the health coach monthly to create a personalized plan for improving brain-healthy self-management behaviors. Data were collected from 22 participants at baseline and 3-month follow-up, which were analyzed using paired-samples t-tests and sign-rank tests. The average age of participants was 76, with 72% female and 95% White. Almost half (45%) reported subjective memory complaints. Compared to baseline, on average, participants reported significant improvements in their mindfulness (t=-1.73, P = 0.001) and nutrition (t=-4.50, P < 0.001), regardless of the chosen focus area for their person brain health goals. Findings suggest the feasibility of a brain health coaching program and the effectiveness at improving mindfulness practices, including mindful eating and implementing a brain-healthy diet. Consistent engagement with certified health and wellness coaches may assist individuals in reducing their risk for cognitive impairment by adopting and adhering to brain-healthy self-management behaviors.

